# lncRNA OGFRP1 functions as a ceRNA to promote the progression of prostate cancer by regulating SARM1 level via miR-124-3p

**DOI:** 10.18632/aging.103007

**Published:** 2020-05-19

**Authors:** Keqiang Yan, Lifang Hou, Tiantian Liu, Wei Jiao, Qian Ma, Zhiqing Fang, Shimin Zhang, Daoqing Song, Jikai Liu, Xinghua Gao, Yidong Fan

**Affiliations:** 1 Department of Urology, QiLu Hospital of Shandong University, Jinan 250012, Shandong, P.R. China; 2 Department of Gynecology, Jinan Central Hospital, Jinan 250012, Shandong, P.R. China; 3 Pathology Department, Basic Medical School, Shandong University, Jinan 250012, Shandong, P.R. China; 4 Department of Urology, Jinan Central Hospital Affiliated to Shandong First Medical University, Jinan 250012, Shandong, P.R. China

**Keywords:** long non-coding RNAs, ceRNA, SARM1, miR-124-3p, prostate cancer

## Abstract

lncRNA can interact with miRNA as a ceRNA to participate in the regulation of target gene expression, thus playing an important role in the development of malignant tumors. In this research, we found that OGFRP1 was up-regulated in prostate cancer (PCa) clinical samples and cell lines. Additionally, OGFRP1 is significantly associated with TNM stages III and IV and perineural invasion. Knockdown of OGFRP1 inhibited the growth of PCa cells, suggesting a promotional effect of OGFRP1 in tumor progression. Interestingly, OGFRP1 primarily localized in the cytoplasm, while miR-124-3p was found to bind to OGFRP1. Therefore, we further analyzed the downstream target of miR-124-3p using TargetScan. The result of the luciferase reporter gene assay displayed that SARM1 was a downstream target of miR-124-3p in two PCa cell lines. The overexpression of SARM1 promoted growth and metastasis in PCa cells. Knockdown of OGFRP1 and overexpression of miR-124-3p markedly restored the promotion of SARM1 to PCa cells. In conclusion, lncRNA OGFRP1 completely bound to miR-124-3p and relieved their inhibition on SARM1, thus promoting the growth of PCa cells. This report extended our understanding of the underlying molecular mechanisms of lncRNAs in PCa, which could help us find novel diagnostic and therapeutic targets.

## INTRODUCTION

Prostate cancer (PCa) is the second leading cause of cancer-related deaths in men living in Western countries, and its incidence is increasing every year [1–3]. Although advanced methods, such as docetaxel chemotherapy and androgen deprivation therapy have been applied for PCa treatment [4, 5], the survival rate of PCa still remains low. Furthermore, 30% of PCa patients experience recurrence after primary therapy [6]. Therefore, exploring the pathogenesis of PCa and finding new therapeutic targets can provide new strategies for diagnosis and accurate treatment of PCa.

Long non-coding RNAs (lncRNAs) refer to non-coding RNAs longer than 200 nucleotides in length. Numerous studies have confirmed that lncRNAs are involved in almost all biological processes [7]. As for tumors, lncRNAs play an important regulatory role in tumor proliferation, survival, metastasis, and so on [8–11]. Recently, the Genetics and Epigenetics Research Laboratory of the University of Texas has demonstrated that lncRNAs are closely involved in regulating various human cancers [12]. The “star molecules” such as MALAT1, HOTAIR, and H19 function as oncogenes or tumor suppressor genes in the malignant development of various cancers. These various cancers include bladder cancer, brain cancer, stomach cancer, kidney cancer, and lung cancer [12]. However, the current understanding of lncRNAs is lacking. According to related statistics, the number of lncRNAs in human cells is approximately 59,000, but only about 10,000 species of lncRNAs have currently been identified by scientists [13, 14]. Moreover, due to low abundance and high tissue specificity, lncRNAs are usually more difficult to study than protein coding genes [15–17]. In summary, lncRNAs are an important emerging research area that not only provide scientists with a vast ocean of gene research but may also bring great insight into the clinical treatment of tumors. OGFRP1 (NCBI ID: 388906), located at chromosome 22q13.2, is a newly identified lncRNA that is 1256bp in length. As far as we know, there is only one study reporting that downregulation of lncRNA OGFRP1 inhibits hepatocellular carcinoma progression by AKT/mTOR and Wnt/β-catenin signaling pathways [18].

In our study, we aim to investigate the role and network of OGFRP1 in the progression of PCa. Our study will provide new insights into the therapeutic application of OGFRP1 in PCa.

## RESULTS

### OGFRP1 was upregulated in human PCa

**Figure 1:**
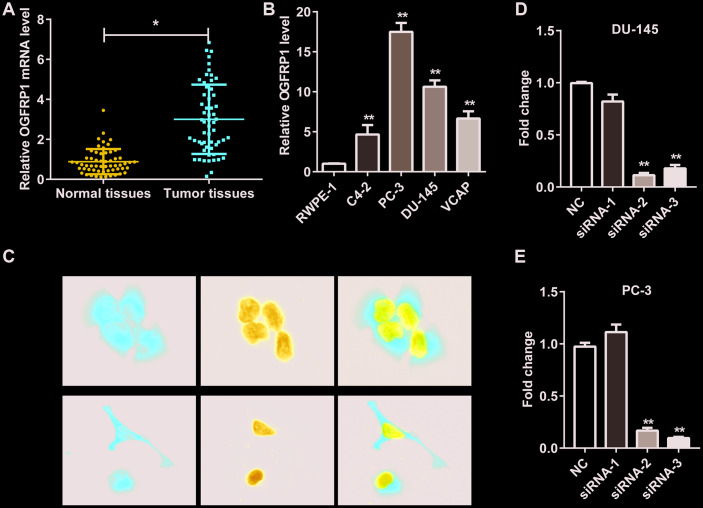
**OGFRP1 was upregulated in PCa and located in the cytoplasm.** (**A**) OGFRP1 levels in 57 pairs of PCa and adjacent tissues were detected by qPCR. (**B**) OGFRP1 was upregulated in PCa cell lines (PC-3, DU-145, C4-2 and VCAP) compared with a normal human prostatic epithelial cell line (RWPE-1). (**C**) Subcellular location of OGFRP1 in DU-145 and PC-3 cells was analyzed using FISH. (**D** and **E**) qPCR demonstrated that siOGFRP1 transfection inhibits mRNA expression of OGFRP1 in both DU-145 and PC-3 cells. SiRNA2 for DU145 and siRNA3 for PC3 were applied in all of the following experiments due to the interference efficiency. All experiments were performed 3 times. *P<0.05; **P<0.01.

First, we detected the expression of lncRNA OGFRP1 in 57 pairs of PCa and adjacent tissues. As shown in Figure 1A, OGFRP1 levels were significantly higher in PCa tissues than in adjacent tissues. In addition, OGFRP1 was upregulated in PCa cell lines (PC-3, DU-145, C4-2 and VCAP) compared to the normal human prostatic epithelial cell line (RWPE-1) (Figure 1B). PC-3 and DU-145 were applied in all of the following experiments due to the higher level of OGFRP1.

### PCa patients with TNM stages III/IV and perineural invasion showed higher OGFRP1 levels

The correlation between OGFRP1 and clinicopathological characteristics of prostate cancer was analyzed. 50% of samples were used as a cut-off between high and low OGFRP1 expression. As shown in Table 1, OGFRP1 was upregulated in PCa patients with TNM stages III and IV compared to PCa patients with stages I or II. Moreover, PCa patients with perineural invasion showed enhanced OGFRP1 expression. These results indicated that OGFRP1 was associated with tumor progression and perineural invasion.

**Table 1:** Correlation between OGFRP1 and clinicopathological characteristics of prostate cancer.

**Characteristics**	**OGFRP1 expression**		***P* value**
	**Low**	**High**	
Age			
≤60	13	16	0.509
>60	15	13	
Serum PSA			
≤10	9	7	0.739
>10	19	12	
TNM stage			
I/II	16	5	0.037*
III/IV	12	14	
Perineural invasion			
Yes	11	19	0.047*
No	17	10	
Gleason score			
≤6	6	9	0.41
>6	22	20	

*P<0.05.

### OGFRP1 was located in the cytoplasm

We investigated the subcellular location of OGFRP1 in DU-145 and PC-3 cells using FISH. As shown in Figure 1C, OGFRP1 was distributed in both the nucleus and cytoplasm. It mainly localized in the cytoplasm, suggesting that it might be involved in the process of epigenetic regulation.

In order to determine the role of OGFRP1 in PCa cells, three candidate siRNAs were conducted and transfected into DU145 and PC3 cells. QRT-PCR results showed that siRNA2 and siRNA3 could efficiently knock down OGFRP1 in both cell lines (Figure 1D). siRNA2 for DU145 and siRNA3 for PC3 were applied in all of the following experiments due to the interference efficiency.

### OGFRP1 decoyed miR-124-3p in PCa cells

**Figure 2:**
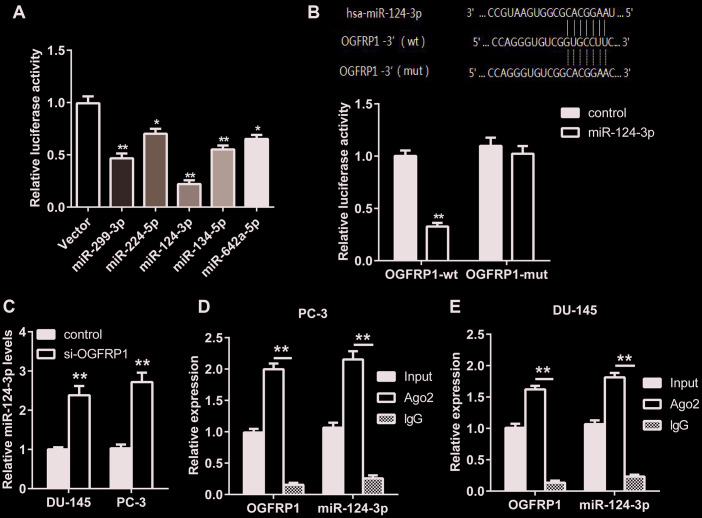
**OGFRP1 decoyed miR-124-3p in PCa cells.** (**A**) Luciferase assay was performed to detect the binding of OGFRP1 to miRNAs (miR299-3p, miR-224-5p, miR-124-3p, miR-134-5p, and miR-624a-5p). (**B**) Luciferase reporter gene assay was performed to confirm the binding of OGFRP1 to miR-124-3p. (**C**) qPCR results proved that OGFRP1 knockdown lead to the up-regulation of miR-124-3p levels. (**D** and **E**) The association of OGFRP1 and miR-124-3p between Ago2 in DU-145 and PC-3 cells was confirmed using RIP. *P<0.05; **P<0.01.

The analysis on TargetScan revealed that five microRNAs (miR299-3p, miR-224-5p, miR-124-3p, miR-134-5p, and miR-624a-5p) had binding sites with the sequence of OGFRP1. We constructed a luciferase vector containing the sequence of OGFRP1 and co-transfected it into 293T cells with mimics of 5 microRNAs, respectively. As shown in Figure 2A, the relative luciferase activity in cells transfected with miR-124-3p decreased significantly, suggesting that it may be a binding target molecule of OGFRP1. Therefore, we validated this hypothesis using a luciferase reporter gene assay. As shown in Figure 2B, the results of the luciferase reporter gene assay proved that the binding of OGFRP1 and miR-124-3p is effective in reducing fluorescence activity, while mutant OGFRP1 and miR-124-3p is not. Moreover, the expression of miR-124-3p declined after the transfection of siOGFRP1 (Figure 2C).

To further verify the mode of action of OGFRP1 and miR-124-3p, a RIP experiment was performed using the Ago2 antibody. As shown in Figures 2D and 2E, both OGFRP1 and miR-124-3p were observed in Ago2 pellets in DU145 and PC3 cells.

### Knockdown of OGFRP1 and mimics of miR-124-3p both inhibited cell growth in PCa cell lines

**Figure 3:**
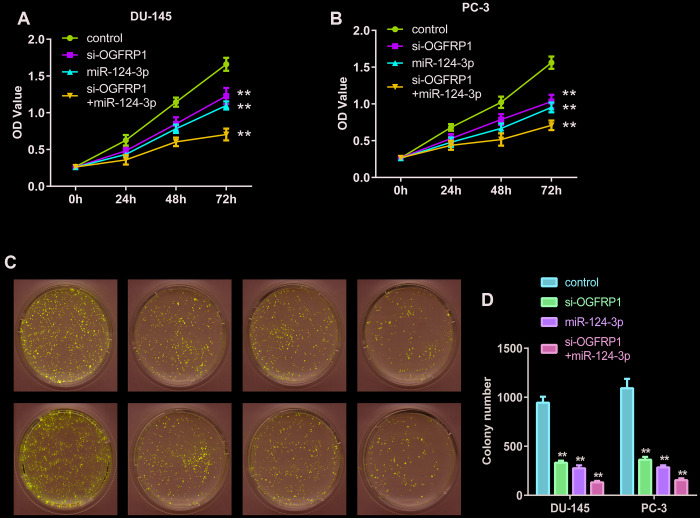
**miR-124-3p and knockdown of OGFRP1 inhibited the proliferation in PCa cells.** (**A** and **B**) Cell proliferation of DU145 and PC3 was analyzed by CCK8 assay, which was detected at different time points, including 0, 24, 48, and 72 h. (**C** and **D**) Clone formation of the DU145 and PC3 cells after transient transfection. **P<0.01.

**Figure 4:**
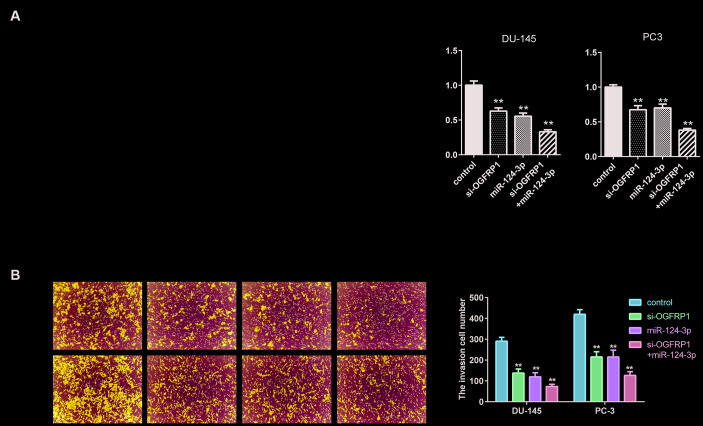
**miR-124-3p and knockdown of OGFRP1 inhibited the migration and invasion in PCa cells.** (**A**) Cell invasion of DU145 and PC3 cells was detected by Transwell assay. (**B**) Cell migration of siOGFRP1 transfected DU145 and PC3 cells was detected by wound healing assay. **P<0.01.

**Figure 5:**
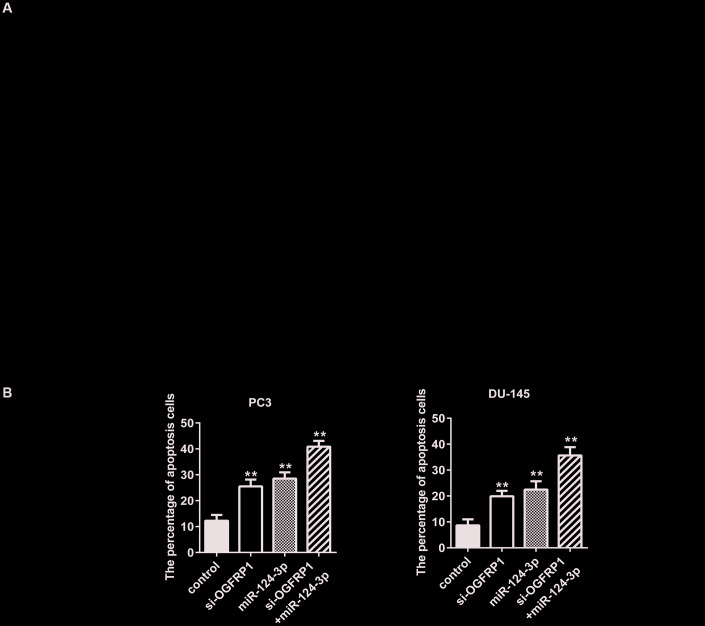
**miR-124-3p and knockdown of OGFRP1 promoted apoptosis in PCa cells.** (**A**) Cell apoptosis of DU145 and PC3 was detected by flow cytometry. (**B**) Analysis of percentage of apoptotic cells. **P<0.01.

We further used CCK8 and clone formation assay to investigate the cell proliferation of DU145 and PC3 cells. As shown in Figure 3A and 3B, cell viability significantly decreased compared with the control group 72 h after transfection with si-OGFRP1 and miR-124-3p. Clone numbers of the si-OGFRP1, miR-124-3p, and si-OGFRP1+miR-124-3p groups decreased in both cell lines (Figure 3C and 3D). These results indicated that OGFRP1 and miR-124-3p played crucial roles in maintaining PCa proliferation. A scratch assay was utilized for evaluating the impact of OGFRP1 knockdown and miR-124-3p on the migration of PCa cells. Wound closure was normalized to the control group, which indicated that cell migration decreased in DU145 and pc3 cells after the transfection of si-OGFRP1 and miR-124-3p (Figure 4A). For transwell invasion assay, the number of cells that invaded Matrigel significantly decreased in the si-OGFRP1, miR-124-3p, and si-OGFRP1+miR-124-3p groups, suggesting that si-OGFRP1 and miR-124-3p both induced the inhibition of cell metastasis in PCa (Figure 4B). Next, we investigated whether OGFRP1 knockdown influenced cell apoptosis in DU145 and PC3 cells. Our results showed that the total percentage of apoptosis significantly increased via si-OGFRP1 and miR-124-3p transfection in DU145 and PC3 cells (Figure 5).

### MiR-124-3p inhibited the expression of SARM1 via binding to its 3’ UTR

**Figure 6:**
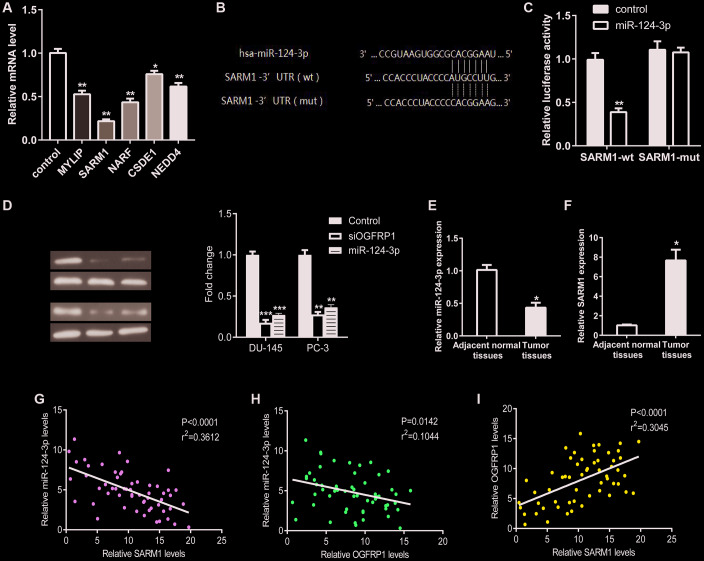
**MiR-124-3p inhibited the expression of SARM1 via binding to its 3’ UTR.** (**A**) The mRNA levels of the predicted target genes (MYLIP, SARM1, NARF, CSDE1, and NEDD4) in PCa cells transfected with miR-124-3p mimics were detected using qPCR. (**B** and **C**) Luciferase reporter gene assay was performed to confirm the binding of miR-124-3p to SARM1. (**D**) The expression of SARM1 was detected by western blot in DU-145 and PC-3 cells. (**E** and **F**) miR-124-3p and SARM1 levels in 57 pairs of PCa and adjacent tissues were detected by qPCR. (**G**–**I**) The correlation between OGFRP1, miR-124-3p, and SARM1 levels was analyzed. *P<0.05; **P<0.01; ***P<0.001.

We analyzed the target mRNA of miR-124-3p using TargetScan. The mRNA levels of the predicted target genes (MYLIP, SARM1, NARF, CSDE1, and NEDD4) in PCa cells transfected with miR-124-3p mimics were detected using qPCR. As shown in Figure 6A, after the transfection of miR-124-3p, the expression of SARM1 decreased significantly. This suggests that it may be a binding target molecule of miR-124-3p. Therefore, we validated the binding of miR-124-3p and SARM1 using a luciferase reporter gene assay. As shown in Figure 6B and 6C, the binding of SARM1’s 3’ UTR and miR-124-3p is effective in reducing fluorescent activity while mutant SARM1’s 3’ UTR are not. In addition, the expression of SARM1 was significantly inhibited by the knockdown of OGFRP1, as well as the transfection of miR-124-3p mimics in both DU-145 and PC-3 cells (Figure 6D). We further detected the expression levels of miR-124-3p and SARM1 in 57 pairs of PCa and adjacent tissues using qPCR. Consistent with our prediction, the miR-124-3p level was downregulated in PCa tissues (Figure 6E) while SARM1 expression was upregulated in PCa tissues compared to the adjacent tissues (Figure 6F). The correlation analysis results demonstrated that miR-124-3p and SARM1 were correlated. In addition, the correlation between miR-124-3p and SARM1 was strong. However, the correlation between miR-124-3p and OGFRP1 was strongly negative with statistical significance (Figures 6G and 6H). In contrast, the expression of OGFRP1 and SARM1 displayed a strong positive correlation and was statistically significant (Figure 6I).

### SARM1 promoted proliferation and metastasis in PCa cells

**Figure 7:**
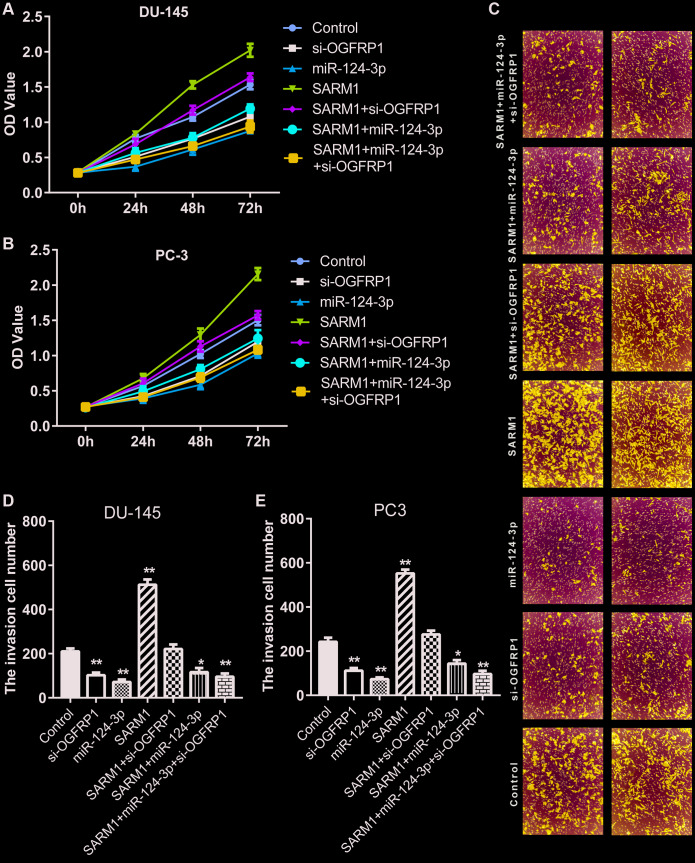
**SARM1 promoted the proliferation and metastasis in PCa cells.** (**A** and **B**) Cell proliferation of DU145 and PC3 was analyzed by CCK8 assay. (**C**–**E**) Cell invasion of DU145 and PC3 cells was detected by Transwell assay. *P<0.05 *vs.* control; **P<0.01 *vs.* control; ^#^*P*<0.05 *vs.* SARM1; ^##^*P*<0.01 *vs.* SARM1; ^&^*P*<0.05 *vs.* si-OGFRP1.

To investigate the role of SARM1 in tumor cells, an overexpression vector of SARM1 was constructed and transfected into DU-145 and PC-3 cells. CCK8 assay proved that the overexpression of SARM1 increased cell viability in both DU-145 and PC-3 cells (Figure 7A and 7B). Transwell results showed that the number of cells that invaded the Matrigel significantly increased in cells transfected with the overexpression vector of SARM1 (Figures 7C–7E). These results proved that SARM1 promoted proliferation and metastasis in PCa cells.

### OGFRP1 functions as a competing endogenous RNA (ceRNA) to promote the progression of prostate cancer

Our data has confirmed that OGFRP1 promoted SARM1 expression as a ceRNA via binding to miR-124-3p. To further support these results, we transfected si-OGFRP1 into SARM1 overexpression cells (SARM1+ si-OGFRP1 group). Meanwhile, miR-124-3p was transfected into SARM1 overexpression cells to generate the SARM1+miR-124-3p group; miR-124-3p and si-OGFRP1 were transfected into SARM1 overexpression cells to generate the SARM1+miR-124-3p+si-OGFRP1 group. Cell proliferation of DU145 and PC3 was analyzed by CCK8 assay. As shown in Figure 7A and 7B, cell proliferation was significantly inhibited in the SARM1+si-OGFRP1 and SARM1+miR-124-3p groups compared with the SARM1 group. Meanwhile, compared with si-OGFRP1, the proliferation of SARM1+si-OGFRP1 was markedly enhanced. Then, cell invasion of DU145 and PC3 cells was detected by Transwell assay. The number of cells that invaded the Matrigel significantly decreased in the SARM1+si-OGFRP1 and SARM1+miR-124-3p groups compared with the SARM1 group. Compared with si-OGFRP1, the number of cells that invaded the Matrigel of the SARM1+si-OGFRP1 group was markedly increased (Figure 7C–7E).

## DISCUSSION

First, we found that OGFRP1 was up-regulated in both PCa clinical samples and cell lines and is significantly associated with TNM stages III and IV and perineural invasion. Knockdown of OGFRP1 inhibited the growth of PCa cells, suggesting a promotional effect of OGFRP1 in tumor progression.

Studies have shown that lncRNA acts as an important regulatory non-coding RNA, mainly involved in the regulation of gene expression by acting as a "signal" molecule or "inducing" molecule in combination with DNA or protein. In recent years, it has been found that lncRNA can interact with miRNA as a ceRNA to participate in the regulation of target gene expression, thus playing an important role in the development of malignant tumors [19]. With the development of sequencing technology and the analysis of the accumulated tumor transcriptomics data, it has been found that more than 10,000 lncRNAs may have potential ceRNAs characteristics [20]. lncRNAs, as a competitive platform for microRNAs and RNA, are involved in the regulation of the cell cycle and cell death in many malignant tumors, such as breast cancer, gastric cancer, liver cancer, lung cancer, and renal cancer. It can also affect the invasion and metastasis of tumors [21, 22]. In 2010, Poliseno et al. confirmed that lncRNA PTENP1 up-regulates the expression of PTEN, a well-known tumor suppressor gene, by adsorbing microRNA-19 and microRNA-20a. As a result, it inhibits the downstream PI3K signaling pathway of PTEN and inhibits cell growth in prostate cancer [23].

In this research, we proved that lncRNA OGFRP1 completely bound to miR-124-3p and relieved their inhibition on SARM1. This caused the expression of SARM1 to be up-regulated, thus promoting the growth of PCa cells. miR-124-3p has a relatively low expression trend in a variety of tumors and plays an important role in tumor proliferation and progression. Studies have shown that miR-124-3p is down-regulated in PCa and is a potential tumor suppressor miRNA that inhibits the proliferation of PCa cells by targeting androgen receptors [24]. Our data proved that SARM1 was a downstream target of miR-124-3p in two PCa cell lines.

SARM1, also known as SAMD2, plays an important role in neurodegenerative diseases, but its role in tumors has not been reported [25]. We found that the overexpression of SARM1 promoted the proliferation, migration, and invasion and inhibited apoptosis in DU-145 and PC-3 cells. Overexpression of SARM1 could also eliminate the inhibition of cell proliferation and metastasis induced by OGFRP1 knockdown or miR-124-3p overexpression in DU-145 and PC-3 cells. This data indicates that overexpression of SARM1 might prevent the regulation of OGFRP1/miR-124-3p on the function of prostate cancer cells. Therefore, we hypothesized that this molecule is a potential target for the treatment of PCa and deserves further study. However, our research is limited to cell lines cultured *in vitro*. The study of the OGFRP1 network should be further verified *in vivo*. Additionally, the application of OGFRP1, miR-124-3p, and SARM1 in the clinical treatment of PCa also needs *in vivo* experiments.

In conclusion, for the first time, we confirmed that OGFRP1 functions as a ceRNA by decoying miR-124-3p to promote the expression of SARM1 in PCa cells. This ceRNA network promoted the proliferation and induced the apoptosis of PCa cells. This report will extend our understanding of the underlying molecular mechanisms of lncRNAs in PCa, which may help find novel diagnostic and therapeutic targets.

## MATERIALS AND METHODS

### Samples and cell lines

57 pairs of PCa tissues and adjacent tissues were collected from PCa patients at the Jinan Central Hospital. Cells used in this research (PCa cell lines: PC-3, DU-145, C4-2 and VCAP; normal human prostatic epithelial cell line: RWPE-1; 293T) were all purchased from the Cell Bank of the Chinese Academy of Sciences (Shanghai, China). Cells were cultured in DMEM and RMPI1640 medium (Gibco BRL) respectively at 37 °C with 5% CO_2_. The medium was combined with 10% fetal bovine serum (FBS, Gibco). Upon entering into the logarithmic phase, cells were transfected using Lipofectamine2000 (Invitrogen). The untreated DU-145 and PC-3 cells were used as the blank control (control group), while DU-145 and PC-3 cells transfected with nonspecific siRNA were used as the negative control (NC group). The sequences used in this research are showed in Supplementary Table 1.

### Quantitative reverse transcription polymerase chain reaction (qRT-PCR)

The RNA was extracted from fresh or frozen samples collected from PCa patients by using RNAlater™ Stabilization Solution (Invitrogen). After transfection for 24 hours, total RNA from cells was extracted using TRIzol reagent (Invitrogen). RNA quality and concentration were quantified using a Nanodrop 2000 system (Thermo Fisher Scientific, Inc.). PrimeScript RT reagent kit (TaKaRa Bio, Shiga, Japan) was used to synthesize cDNA from 1 μg of total RNA. Then, RT-PCR was performed using a SYBR Premix Ex Taq™ kit (TaKaRa Bio) on an ABI Prism 7500 Sequence Detection System (Applied Biosystems, Foster City, CA, USA). β-actin expression was used as the internal control. The relative gene expression was calculated using the 2^−ΔΔCt^ method. For the detection of miRNA, reverse transcription PCR was performed using a miRNA cDNA Synthesis Kit (CoWin Biotech, Beijing, China), and qPCR was performed using a miRNA qPCR Assay Kit (CoWin Biotech, Beijing, China). The used primers are showed in Supplementary Table 2.

### Fluorescence in situ hybridization (FISH)

FISH probes were designed and synthesized by RiboBio, Co., Ltd (Guangzhou, China). FISH assay was performed using the Fluorescent In Situ Hybridization Kit (Ribo, Guangzhou, China) according to the manufacturer's instructions. Fluorescence images were obtained using a confocal microscope (Leica).

### Luciferase reporter gene assay

The OGFRP1 wildtype and mutant reporter plasmids were constructed for the luciferase reporter gene assay. A OGFRP1 sequence/sterile alpha and TIR motif containing 1 (SARM1) 3’ UTR fragment, which contained the binding cite predicted by TargetScan, was recombined into a pGL3.0 vector to generate the OGFRP1/SARM1-wt plasmid. Then, a mutated OGFRP1/SARM1 3’ UTR fragment was recombined into the luciferase construct to generate the OGFRP1/SARM1-mut plasmid. Cells that were transfected with pCMV-miR-124-3p were considered the miR-124-3p overexpression group, while cells transfected with pCMV-miR empty vector functioned as the control group both with the co-transfection of Renilla luciferase expression vector. The luciferase expression was determined by the Dual-Glo Luciferase Assay System (Promega, USA) and normalized to the wild-type control group.

### RIP based on Ago2

Molecular binding was verified using a Magna RIP™ RNA-Binding Protein Immunoprecipitation Kit (Millipore, MA). DU-145 and PC3 cells were used to perform RIP assay according to the manufacturer's instructions. qPCR was used to detect the expression of OGFRP1 and miR-124-3p.

### Cell proliferation analysis

### CCK8 assay

PCa cells were planted into a 96-well plate at nearly 3000 per well. 10 μl CCK8 solution was added to each well for 24 h, 48 h, and 72 h, then incubated for 2 h. The OD value at 450 nm was measured as cell viability.

### Clone formation assay

PCa cells were transiently transfected and seeded on a 6-cm dish at a total number of 200-300. The cells were cultured until clones were visible by the naked eye and then stained with 0.1% crystal violet for 20 min. Afterwards, the results were recorded with the ChemiDoc XRS imaging system.

### Wound healing and matrigel invasion assays

For wound healing assays, cells were seeded in a 6-well plate and maintained to 90% confluence. A scratch was then created with a 200 μL pipette tip on a cell monolayer. After washing detached cells with PBS, cells were cultured for another 24 h. The wound width was measured at 0 h and 24 h. Cell migration velocity was represented by wound closure that was normalized to that of the control group.

For Matrigel invasion assays (Millipore), 1×10^6^ cells distributed in 500 μL of serum-free medium were added to Transwell inserts. 500 μL medium containing 10% FBS was added to the bottom chamber. After 24 h, cells invaded the bottom side of the membrane and then were stained and counted under a microscope.

### Cell apoptosis analysis

DU145 and PC3 cells were transfected and harvested after 48 h. For cell apoptosis analysis, cells were sequentially washed with PBS and incubated with the Annexin V-FITC and PI for 15 min in the dark. All cell samples were analyzed by using the BD Biosciences FACSCalibur flow cytometer (BD Biosciences) and FlowJo10 software.

### Statistical analysis

All data was represented from three independent experiments. GraphPad Prism 7.0 and SPSS 18.0 software were used for all statistical analyses. Correlation between OGFRP1 levels and clinicopathological characteristics was analyzed with a *χ^2^* test. The difference analysis between the two groups was compared using a one-way ANOVA. Correlation between the mRNA expression levels of genes was analyzed by regression analysis with Pearson’s correlation coefficient. *P*<0.05 was considered statistically significant.

## Supplementary Material

Supplementary Tables
