# Supplementary material for: lncRNA OGFRP1 functions as a ceRNA to promote the progression of prostate cancer by regulating SARM1 level via miR-124-3p
Source: Aging (Albany NY). 2020 May 19;12(10):8880–92. doi: 10.18632/aging.103007 (PMC7288971; doi:10.18632/aging.103007)
Supplement: Supplementary Tables [file aging-12-103007-s001..pdf]

SUPPLEMENTARY TABLES

Supplementary Table 1. Sequences used in this research.

Name	Sequence
OGFRP1-siRNA1	5'-GGTGTTCACATGGCAGTAA-3'
OGFRP1-siRNA2	5'-GGATACTGAGAGTGCACAA-3'
OGFRP1-siRNA3	5'-GCATTGACATGTTTGGCAT-3'
siNC	5'-UUCUCCGAACGUGUCACGUTT-3'
miR-299-3p	5'-UAUGUGGGAUGGUAAACCGCUU-3'
miR-224-5p	5'-CAAGUCACUAGUGGUUCCGUU-3'
miR-124-3p	5'-UAAGGCACGCGGUGAAUGCC-3'
miR-134	5'-UGUGACUGGUUGACCAGAGGGG-3'
miR-642a-5p	5'-GUCCCUCUCCAAAUGUGUCUUG-3'
U6	5'-CTCGCTTCGGCAGCACA-3'

Supplementary Table 2. Primers used in this research.

Name	Sequence
OGFRP1-F	5'-AAGATGAAGCTGCGGAGTTG-3'
OGFRP1-R	5'-CCAGATTAGGATGCCACACTTC-3'
β-actin-F	5'-GAACCCTAAGGCCAAC-3'
β-actin-R	5'-TGTCACGCACGATTTCC-3'
MYLIP-F	5'-ACGGTCACCAAGGAATCTGGGA-3'
MYLIP-R	5'-CCTTCAAGTCACGGCTATACTGC-3'
SARM1-F	5'-CCAGATGTCTTCATCAGCTACCG-3'
SARM1-R	5'-CACTCTGGATGAGTTTGTCCTCG-3'
NARF-F	5'-GCTGTTCAACGAGGATGTGGAG-3'
NARF-R	5'-CAGCAAAGCGTAACACCACCTC-3'
CSDE1-F	5'-GTAGTTTGTGCCATGAAGGAGGC-3'
CSDE1-R	5'-CCACATCATCGCCAGGCTGTAA-3'
NEDD4-F	5'-CAGAAGAGGCAGCTTACAAGCC-3'
NEDD4-R	5'-CTTCCCAACCTGGTGGTAATCC-3'
